# Hsa-miR-15a and Hsa-miR-16-1 Expression Is Not Related to Proliferation Centers Abundance and Other Prognostic Factors in Chronic Lymphocytic Leukemia

**DOI:** 10.1155/2013/715391

**Published:** 2013-12-12

**Authors:** Maura Rossi, Fabio Fuligni, Maria Ciccone, Claudio Agostinelli, Simona Righi, Marco Luciani, Maria Antonella Laginestra, Gian Matteo Rigolin, Maria Rosaria Sapienza, Anna Gazzola, Claudia Mannu, Antonio Cuneo, Stefano Pileri, Pier Paolo Piccaluga

**Affiliations:** ^1^Department of Experimental, Diagnostic, and Specialty Medicine, Bologna University Medical School, Unit of Hematopathology, S. Orsola-Malpighi Hospital, Via Massarenti 9, 40138 Bologna, Italy; ^2^Department of Biomedical Sciences, Hematology Section, S. Anna Hospital, University of Ferrara, Via Aldo Moro 8, Cona, 44124 Ferrara, Italy

## Abstract

Chronic lymphocytic leukemia/small lymphocytic lymphoma (CLL) is the commonest leukemia in adults. Here, we aimed to evaluate hsa-miR-15a/hsa-miR-16-1 expression in CLL tissues by qPCR and correlate it with the other clinicopathological features and clinical outcome. 40 formalin-fixed paraffin-embedded (FFPE) lymph node samples obtained from CLL/SLL patients were classified into two categories, “PCs rich” and “typical.” We found a significant common expression level of 4 miRNAs; however, we did not find any significant relationship between PCs presence and miRNAs expression. Moreover, neither the presence of 13q deletion nor the percentage of cells carrying the deletion strictly correlated with miRNAs expression levels, although a significant number of patients with 13q deletion presented hsa-miR-16-1-3p levels below the median value in normal samples (*P* < 0.05). Finally, although no correlation was found between the expression of each miRNA and other clinicopathological features (Ki67, CD38, ZAP70, and IGVH@ hypermutations), the OS curves showed a positive trend in patients with miRNAs downregulation, though not statistically significant. In conclusion, we showed for the first time that all miRNAs can be successfully studied in FFPE CLL tissues and that del13q and PCs richness do not strictly correspond to miRNAs downregulation; therefore, a specific evaluation may be envisaged at least in patients enrolled in clinical trials.

## 1. Introduction 

Chronic lymphocytic leukemia/small lymphocytic lymphoma (CLL/SLL) is a neoplasm composed of monomorphic small, round to slightly irregular B lymphocytes that accumulate in the peripheral blood (PB), bone marrow (BM), spleen, and lymph nodes [1]. At immunophenotype, CLL/SLL cells usually coexpress CD5 and CD23, weak surface IgM/IgD, CD20, CD22, CD19, CD79a, CD43, and CD11c (weak). CD10 is negative and FMC7 and CD79b are usually negative or weakly expressed in typical cases [1, 2].

A characteristic histopathologic feature of CLL/SLL is represented by the so-called proliferation centers (PCs), which consist of regularly distributed pale areas with numerous prolymphocytes and paraimmunoblasts, resulting in a pseudofollicular pattern [1, 2]. The PCs may promote the selection and the clonal expansion of B cells with a T-dependent immune response to an unknown antigen [3]. However, the significance of abundant PCs remains controversial. Some papers demonstrated that the size of PCs and the amount of paraimmunoblasts in lymph node sections did not show a correlation with clinical course, [4, 5] while others reported the presence of more extensive PCs in follow-up biopsies compared with diagnostic ones [6].

However, although most studies on CLL biology have been carried on Peripheral Blood samples, recent evidence suggested that tissue infiltrating cells and specially those forming the PCs actually represent the reservoir of neoplastic cells and may present with peculiar features, different from Peripheral Blood cells. Recently, Ginè et al. [7] referred that presence of expanded and/or highly active PCs identified a group of patients with “accelerated” chronic lymphocytic leukemia characterized by an aggressive clinical behaviour. Similarly, our group recently showed that the presence of PC-rich samples confers a worse prognosis independently of the other conventional biomarkers [8].

In this regard, as the clinical behavior is quite variable, several biological prognostic factors have been studied in the last decade including mutational status of IGVH genes, the presence or absence of ZAP70 and CD38 at immunophenotyping, and *TP53* mutations [9–18].

In particular, the worst prognosis is correlated with *TP53* mutations, nonmutated IGVH@ genes, and the simultaneous presence of ZAP70+ and CD38+. In addition, recent studies based on high throughput sequencing indicated novel genetic events possiblely associated with disease aggressiveness [19, 20], including *BIRC*, *SF3B1*, and *NOTCH1*. Further, cytogenetic abnormalities detected represent a major for prognostic factor.

About 80% of cases show cytogenetic abnormalities detected by FISH [17]. The most frequent aberrations include 13q14 deletion (40–50% of cases), 11q22-23 deletion (10–20% of cases), total or partial trisomy 12 (10–15% of cases), and 17p13 deletion (2–7% of cases), identifying specific disease subtypes in CLL/SLL [16–18].

CLL with the 13q14 deletion shows a relatively favourable outcome if compared to the other chromosomal aberrations. In particular, 13q14 deletion contains a region called Minimal Deleted Region (MDR), a region of approximately 30 kb that contains *DLEU2* gene, part of *DLEU1*, and the cluster of two miRNAs, namely, hsa-miR-15a and hsa-miR-16-1. Several studies in CLL and in solid tumors have suggested that these miRNAs may be hotspots in neoplastic transformation, being affected by deletion or downregulation in cancer cells [21–29], with their expression levels being also related to the clinical outcome.

Recently, a few papers studied the correspondence between 13q14 deletion and expression of hsa-miR-15a and hsa-miR-16-1 cluster [30–33]; of interest, by studying fresh cells from peripheral blood, the authors failed to find any relevant correlation between the deletion and miRNA expression, indicating possible additional mechanism of regulation, such as epigenetic ones [34–36].

In this study, we aimed to assess for the first time the expression patterns of hsa-miRNA 16-1 and hsa-miR-15a cluster in tissue sample of CLL/SLL and to identify possible correlations with the presence of abundant PCs and other prognostic parameters.

## 2. Materials and Methods

### 2.1. Patients/Samples

A total of 40 lymph node samples obtained from CLL/SLL patients diagnosed according to NCI criteria [37] were included in this study. The patients were referred for histological diagnosis at the Hematopathology Unit of S. Orsola-Malpighi Hospital, Bologna University, Bologna, Italy, between 2002 and 2008. The only selection criterion was represented by the availability of sufficient amount of formalin-fixed paraffin-embedded (FFPE) tissue. The cases were classified into two categories, “PCs rich” and “typical,” according to previously reported criteria [5]. The former included those cases with confluent PCs whereas the latter showed scattered, small, ill-defined PCs in a monotonous background of small, relatively round lymphocytes. As far as the cytogenetic is concerned, 20 samples with 13q deletion and 20 samples without 13q deletion were studied. The main clinicopathological features of these patients are summarized in Tables 1 and 2.

Further, ten reactive FFPE nonneoplastic lymph nodes were used as control.

### 2.2. RNA Extraction

Total RNA was extracted from five 10 *μ*m section of FFPE tissues and treated with DNase using RecoverAll kit according to the manufacturer's instructions (Ambion, USA). Amount and quality of RNA were evaluated measuring the OD at 260 nm and the 260/230 and the 260/280 ratios by Nanodrop (ND-1000 Celbio, Italy).

### 2.3. miRNA Expression

For each RNA sample, 10 ng of total RNA was reverse transcribed using the Taqman MicroRNA reverse transcription kit and gene-specific stem-loop primers for each miRNA (hsa-miR-16-5p, hsa-miR-16-1-3p, hsa-miR-15a-5p, and hsa-miR-15a-3p), named according to miRBase, in conformity with manufacturer's instructions (Applied Biosystems, Applera, Italy). Real-time PCR was performed using Taqman probes specific for each miRNA and for RNU6B, with the latter being used as an endogenous control (Applied Biosystems, Applera, Italy). Real-time PCR was performed using an Applied Biosystems 7900HT Fast Real-Time PCR System.

### 2.4. Immunohistochemistry on Tissue Microarrays

A Giemsa-stained slide was prepared from each paraffin block, and representative tumor regions including scattered or confluent PCs were morphologically identified and marked on every slide. Tissue cylinders with a diameter of 1.0 mm were punched from the marked areas of all blocks and brought into a recipient paraffin block using a precision instrument, as previously described [38]. Tissue microarrays (TMAs) were prepared to perform immunohistochemistry and fluorescence in situ hybridization FISH.

We studied the expression of ZAP-70, CD38, and Ki-67 by immunohistochemistry (IHC) on TMAs containing the 40 cases (in duplicate cores) studied for microRNA expression. 1.5 *μ*m thick sections were cut from each recipient block and tested with anti-ZAP70 (Upstate, Millipore, Billerica, MA, USA, clone 2F3.2: dilution 1 : 80), anti-CD38 (Novocastra, Menarini Diagnostics, Grassina, Italy, clone SPC32: dilution 1 : 80), and anti-Ki-67 (Dako, Glostrup, Denmark, clone Mib-1: dilution 1 : 100). The sections underwent antigen retrieval in PTLink (5 min at 92°C) in EnVision Flex Target Retrieval high PH Solution, and Dako REAL Detection System Alkaline Phosphatase/RED were used. Each evaluation was performed by at least two expert hematopathologists (CA, ES, FB, PPP, and SAP).

### 2.5. Fluorescence In Situ Hybridization

4 *μ*m thick sections were preferred for FISH assays. Each TMA was submitted to hybridization using the Spectrum orange LSI D13S25 DNA probe (band 13q14.3) and Spectrum orange LSI p53 probe (band 17p13.1) (Vysis Co. (Downers Grove, IL, USA), distributed by Abbott Co (Rome, Italy)) in dual-colour hybridization tests using a chromosome-10-centromeric probe as internal control in each experiment; the protocol was made as previously suggested. Further details on FISH analysis have been previously published [8].

Evaluation of FISH results was performed on a fluorescence-equipped microscope (Nikon Italia, Milan, Italy) with a charge-coupled black and white camera and appropriate hardware and software. The results of FISH labeling were interpreted and scored by one investigator unaware of the histological data. Depending on the amount of cells for each core section, at least 100 and usually 200–300 nuclei were manually scored. To ensure accuracy of the analysis, scoring was performed on almost the entire core section, and both cores were screened for each case. Signal screening was performed in those areas without excessive overlapping, avoiding areas where nuclei borders were not clearly shaped.

To establish the cutoff, 12 cases of reactive tonsil, spleen, thymus tissues, and reactive lymph nodes were studied; the cutoff point for positivity for detecting 13q14 deletion and 17p deletion was set at 43%, corresponding to the mean false-positive values obtained on the 12 control cases plus 3 standard deviations [8]. A case was classified as carrier of deletion provided that a concomitant normal hybridization pattern was observed with the chromosome-10-centromeric probe.

### 2.6. Immunoglobulin Heavy Chain Variable Region (IGVH@) Mutational Status

Genomic DNA was isolated from paraffin-embedded lymph nodes using QIAmp DNA mini kit (Qiagen, Milan, Italy) and subjected to amplification of the VDJ (variable/diversity/joining) gene rearrangements by polymerase reaction (PCR). In particular, IGHV rearrangements were amplified with family-specific primers hybridizing to sequences in the framework region 1 or framework region 2 in conjunction with JH primers, in separate reactions for each VH family [39]. IGHV sequences were aligned to the international ImMunoGeneTics database (IGMT http://www.imgt.org/; initiator and coordinator: Marie-Paule Lefranc, Montpellier, France) using the IgBLAST software (http://www.ncbi.nlm.nih.gov/igblast/). IGHV sequences were considered mutated if deviation from the corresponding germline gene was >2%.

### 2.7. Statistical Analysis

Statistical analysis was performed using IBM SPSS Statistics 20.0, using 2^−ΔΔCt^ expression values for each miRNA.

miRNA differentially expressed between different groups (e.g., PCs rich versus typical, with versus without 13q deletion, etc.) was identified using a two-tailed Student's *t*-test for independent samples. The same statistical approach was used to evaluate differences in the expression profile between different pairs of miRNAs.

Further, to assess possible up- or downregulation of single miRNA in each individual case, the expression values were then compared with the threshold value 1 (obtained from 2^−ΔΔCt^ calculation of nonneoplastic samples), applying a 2-tailed single sample *t*-test. Values higher than threshold 1 and statistically significant were classified as upregulated; those lower than threshold 1 and statistically significant were classified as downregulated; Fisher Exact Test on a  2 × 2  contingency table was then used to find significant changes in miRNA regulation (up or down) for different groups (with versus without 13q deletion). The relation between miRNA expression and percentage of cells carrying del13q and between miRNA expression and prognostic factors (Ki67, CD38, and ZAP70) was estimated with Pearson's correlation (*r*) and coefficient of determination *R* ^2^ for linear regression. Overall survival (OS) was calculated from the time of diagnosis to death or last followup. Survival data were analyzed by using the Kaplan-Meier method and the log-rank Mantle-Cox method. Statistical differences were regarded as significant for  *P* value minor or equal to 0.05.

## 3. Results

### 3.1. miRNAs Expression Was Not Related to Proliferation Center Distribution

**Figure 1:**
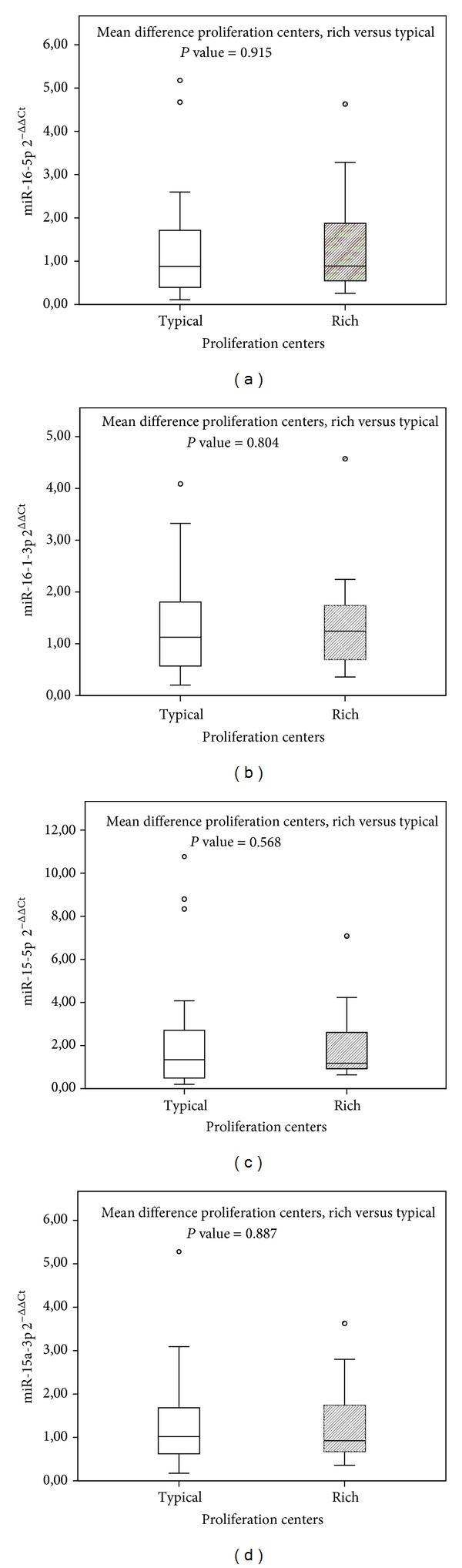
The box plots show the distribution of 2^−ΔΔCt^ values for each miRNA in two different groups (PCs rich and typical). On the  *y*-axis, gene expression quantification values are reported. In the box plots horizontal bars represent median expression values, central rectangles span interquartile range, and dots constitute outlier values. Differences among mean values were calculated by *t*-test. The quantification of miRNAs [hsa-miR-16-5p (a), hsa-miR-16-1-3p (b), hsa-miR-15a-5p (c), and hsa-miR-15a-3p (d)] is not tightly associated with PCs rich and typical CLL samples.

First, we searched for a possible significant relations between the expression of each miRNA and the abundancy of proliferation centers (PCs rich and typical). We did not find any significant relationship between PCs presence (PCs rich and typical) and miRNAs expression. Particularly, hsa-miR-16-5p expression was 1.32 for typical and 1.36 for PCs-rich cases (*P* value = 0.915, mean difference = −0.0432, 95% C.I. from −0.8765 to 0.7879); hsa-miR-16-1-3p expression was 1.32 for typical and 1.40 for PCs-rich cases (*P* value = 0.804, mean difference = −0.0806, 95% C.I. from −0.7259 to 0.5646); hsa-miR-15a-5p expression was 2.41 for typical and 1.98 for PCs rich cases (*P* value = 0.568, mean difference = 0.4250, 95% C.I. from −1.2118 to 2.0618); hsa-miR-15a-3p expression was 1.33 for typical and 1.28 for PCs-rich cases (*P* value = 0.887, mean difference = −0.0454, 95% C.I. from −0.6250 to 0.7159) (Figure 1).

### 3.2. miRNAs Expression Was Not Related to Other Clinicopathological Features

We then looked for possible correlations between the expression of each miRNA and the principal clinical-prognostic factors, including Ki67, CD38, ZAP70, IGVH@ mutations, and 17p deletion. Again, we found no significant correlations for any parameter (not shown).

### 3.3. The Quantification of miRNAs Is Not Tightly Associated with the Presence of 13q Deletion

**Figure 2:**
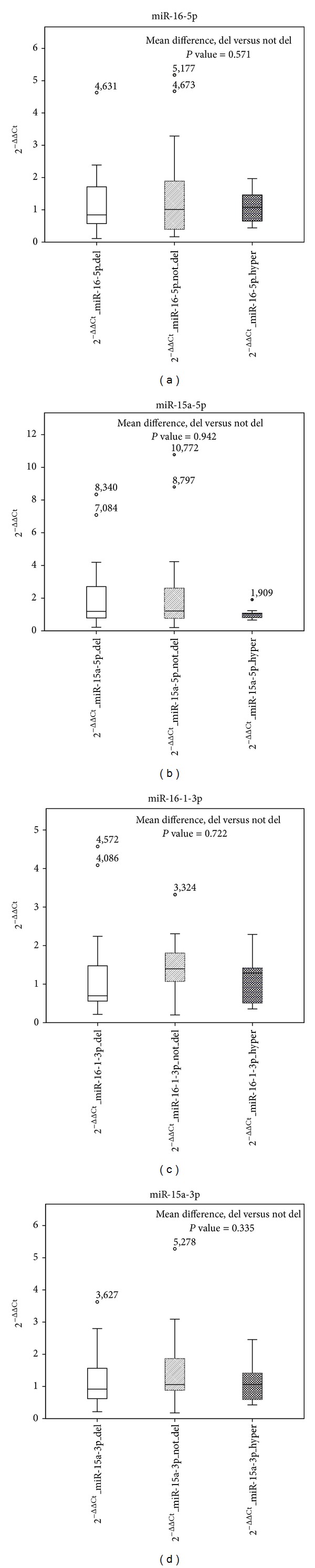
The box plots show the distribution of  2^−ΔΔCt^  values for each miRNA in all the conditions (cases with or without 13q deletion, hyperplasia). On the  *y*-axis, gene expression quantification values are reported. In the box plots horizontal bars represent median expression values, central rectangles span interquartile range, and dots constitute outlier values. Differences among mean values were calculated by *t*-test. The quantification of miRNAs (hsa-miR-16-5p, hsa-miR-15a-5p, hsa-miR-15a-3p, and hsa-miR-16-1-3p) is not tightly associated with the presence of 13q deletion.

Thereafter, based on the previous observation carried on PB samples [28–32] we specifically investigated whether the presence of 13q deletion corresponded to miRNA 15a/16-1 cluster downregulation in our tissue samples. We did not find any significant differences between cases with or without deletion. In particular, the mean expression value of hsa-miR-16-5p was 1,23 for cases carrying 13qDel and 1,44 for cases without deletion (*P* value = 0.571); hsa-miR-16-1-3p expression was 1.22 for cases with deletion and 1.48 for cases without deletion (*P* value = 0.722); hsa-miR-15a-5p expression was 2.21 for cases with deletion and 2.27 for cases without deletion (*P* value = 0.942); hsa-miR-15a-3p expression was 1.16 for cases with deletion and 1.46 for cases without deletion (*P* value = 0.335). Indeed the expression value in both groups was not different from those recorded in nonneoplastic lymph nodes (Figure 2).

**Figure 3:**
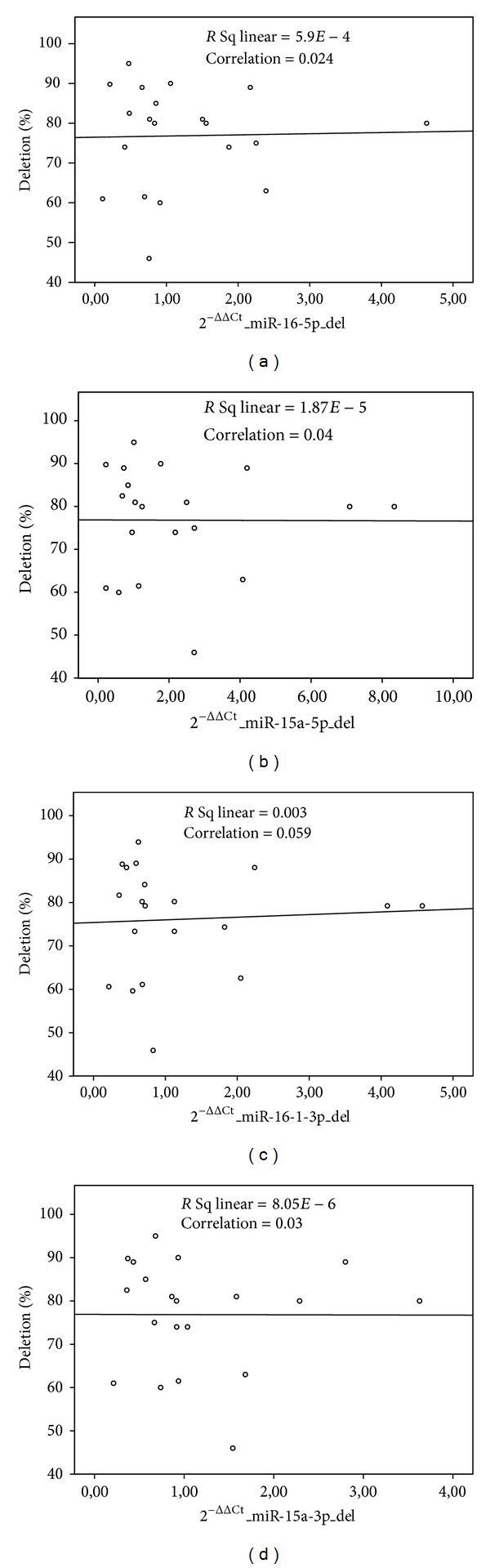
The scatterplots show the distribution of the percentage of deletion on different miRNA's 2^−ΔΔCt^. The percentage of the cells with 13q deletion does not correlate with the expression of miRNAs hsa-miR-16-5p, hsa-miR-15a-5p, hsa-miR-15a-3p, and hsa-miR-16-3p. Pearson correlation coefficient (*r*) and coefficient of linear regression (*R* ^2^) had value close to zero for all miRNAs.

Subsequently, in order to exclude a possible puzzling effect of eventual subclonal heterogeneity, we tried to assess whether miRNAs expression in each case was related to the percentage of cells carrying 13q deletion. Again, we failed to identify any significant correlation, with the miRNA expression levels being independent of the percentage of cells carrying the genetic lesion (Figure 3).

Afterwards, we explored the degree of correlation among the different miRNAs.

**Figure 4:**
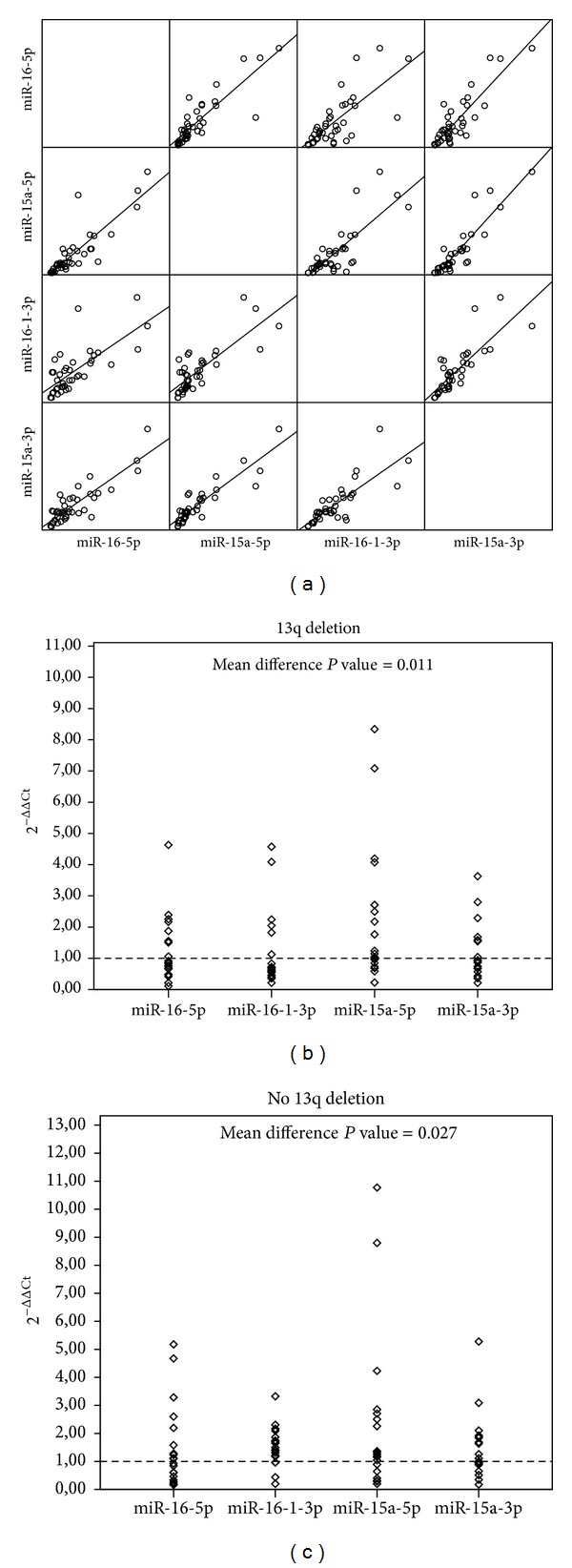
(a) In scatterplot matrix of 2^−ΔΔCt^ values, each pair of miRNA is plotted against each other. Expression values distribution and regression lines show strong positive linear correlation for every couple of miRNA. (b)-(c) Distribution of 2^−ΔΔCt^ for different miRNAs in cases with and without 13q deletion. The expression of miRNAs is variable in cases with and without 13q deletion; the intensity of hsa-miR-15a-5p expression is higher than that of other miRNAs. Mean difference between hsa-miR-15a-5p and hsa-miR-16-5p is statistically significant in both groups of cases with and without 13q deletion.

We found a significant correlation between miRNAs belonging to the same stem loop (hsa-miR-16-5p versus hsa-miR-16-1-3p,  *P* < 0.001; hsa-miR-15a-5p versus hsa-miR-15a-3p,  *P* < 0.001). Furthermore, we found a significant correlation among miRNA of different stem loop, although the degree of correlation was lower than that within each stem loops (Table 3; Figure 4(a)). Such observation was confirmed in both cytogenetic subgroups (13q Del positive or negative) (Figures 4(b) and 4(c)). The significant differences between miR-16-5p and miR-15a-5p could reflect the variability of single molecule expression, but these may also reflect the presence of different mechanism of regulation (i.e., epigenetic mechanism, etc.).

**Figure 5:**
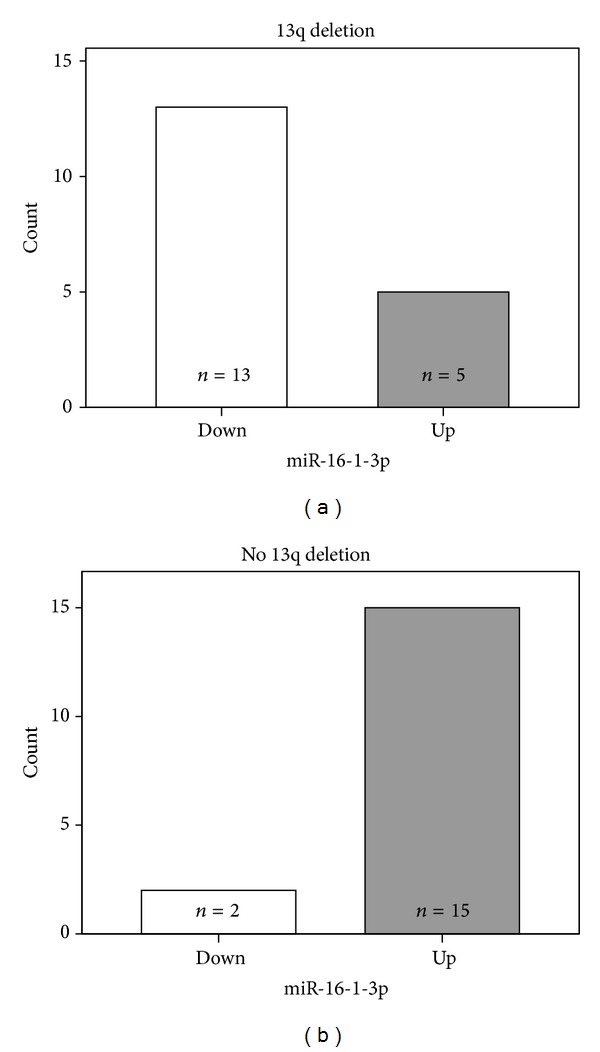
The histogram shows the number of up- and downregulated samples for hsa-miR-16-1-3p cases with and without 13q deletion. A significant number of the patients with 13q deletion presents downregulation of hsa-miR-16-1-3p.

As mentioned above, miRNAs expression did not significantly differed in tumor samples and nonneoplastic tissues (Figure 2). However, when we challenge the miRNAs expression of singular CLL cases versus the median value recorded in reactive lymph node, we observed a significant higher occurrence of downregulation of hsa-miR-16-1-3p in cases carrying 13q deletion (Fisher Exact Test, *P* value < 0.001; Figure 5; Supplementary Table  1 available online at http://dx.doi.org/10.1155/2013/715391).

Together, these data indicate a global concordance in the regulation of two miRNAs independent clusters and confirmed that also in tissues as in PB there is no significant correlation between their expression and the presence of 13qDel, though hsa-miR-16-1-3p was more commonly reduced in presence of the lesion.

### 3.4. The Presence of miRNAs Tended to Correlate with the Clinical Outcome in terms of OS

Finally, as miRNAs expression has been previously associated with patients survival [28], we tested whether it could be related to disease progression/transformation and overal survival (OS) in our series as well.

**Figure 6:**
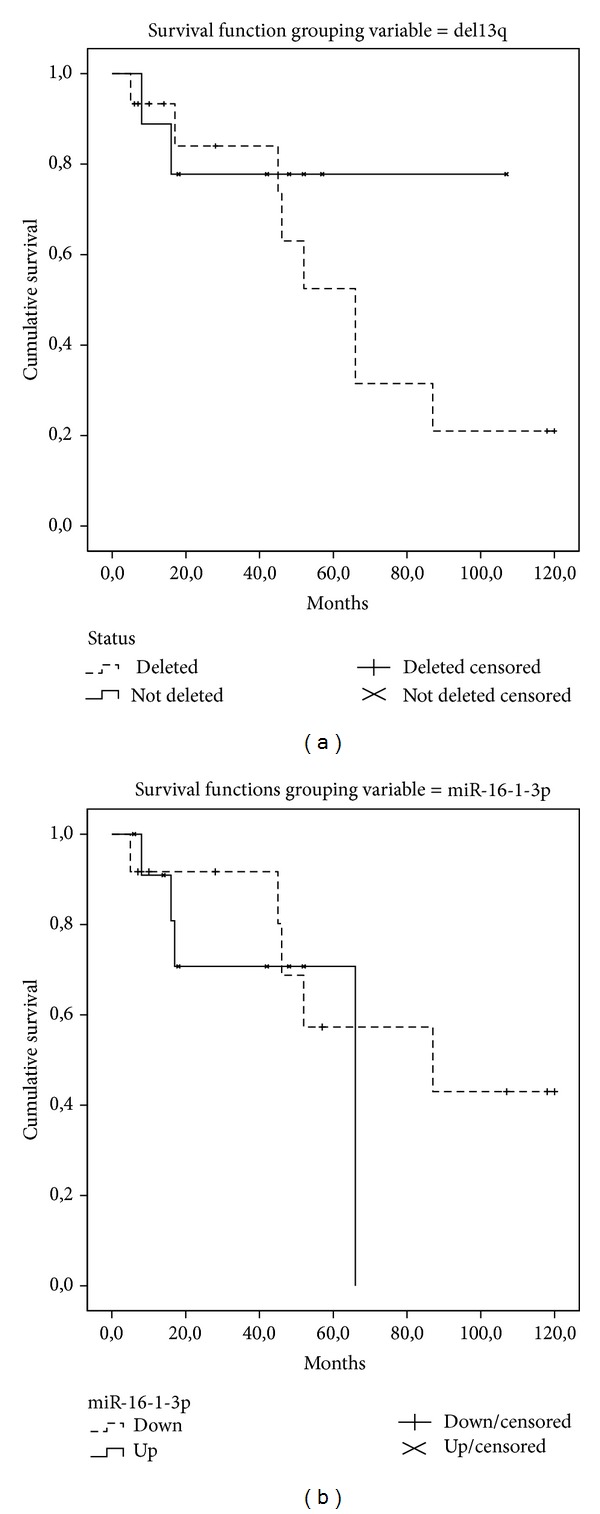
The presence of 13q deletion and the regulation of miR-16-1-3p followed the same trend of correlation with the clinical outcome in term of overall survival although the difference was not statistically significant.

Though a trend in favor of cases with lower expression of hsa-miRNA-16-1-3p was observed, the difference was not statistically significant (median overall survival for up- and downregulation, respectively: 66 months (95% C.I. ranging from 47 to 85 months) and 87 months (95% C.I. ranging from 3 to 170 months), *P* value Logrank Mantel-Cox 0.358). Conversely, the prognostic value of 13q deletion was confirmed (Figure 6).

## 4. Discussion

Chronic lymphocytic leukemia/small lymphocytic lymphoma (CLL/SLL) is the commonest B-cell clonal lymphoproliferative disorder in adults in western countries [1]. Recurrent molecular cytogenetic abnormalities may identify specific disease subtypes in CLL/SLL, with the most frequent aberrations being 13q14 deletion (40–50% incidence). This region includes hsa-miR-16-1 and hsa-miR-15a clusters [22–24].

Beside cytogenetics, the most important additional prognostic factors in CLL are CD38 expression, ZAP70 expression, and IGVH@ mutational status, *TP53* [17], while novel somatic mutations were identified by high throughput sequencing on currently undergoing further clinical evaluation [19, 20, 40]. All of them can be easily and reliably assessed on PB. However, other important disease features need to be evaluated on tissue samples, including the presence of PCs.

Our group in 2011 [8] published that the median survival was significantly lower in presence of a PC-rich pattern, that turned out to be the only predictive factor of an inferior survival at multivariate analysis. In this paper, for the first time, we studied the possible correlation between the high levels of PCs and the expression of hsa-miR-16-5p, hsa-miR-15a-5p, hsa-miR-16-1-3p, and hsa-miR-15a-3p, evaluated in FFPE lymph node samples from patients with CLL.

First, our results showed that the presence of high levels of PCs did not correlate with the expression levels of these miRNAs. This may suggest that miRNA expression is relatively stable in CLL population not being really related to the proliferation rate.

Moreover, our results indicated that neither the presence of 13q deletion nor the percentage of cells carrying the deletion in a given sample strictly correlated with the expression levels of hsa-miR-16-5p, hsa-miR-16-1-3p, hsa-miR-15a-5p, or hsa-miR-15a-3p. This finding, anyway, is not completely surprising, as similar results were reported when PB samples were tested [29, 30, 32]. In this regard, it should be noted that 13q deletion is monoallelic in about 75% of cases [23]. Therefore, compensation by the not-deleted allele could explain the regular expression level of miRNAs even in cases actually carrying the deletion. Of note, monoallelic and biallelic deletion cannot be distinguished by FISH on tissues due to technical reasons (truncation nuclei, etc.); this partially limits the information that can be obtained with this approach. On the other hand, in some wild type (without 13q deletion) cases a reduced expression of miRNAs was observed. This phenomenon could be explained by epigenetic mechanisms, that have been, in fact, involved in the regulation of these miRNAs [35, 36]. In our series, unfortunately, we could not verify this hypothesis due to the lack of residual material.

Further, beside epigenetics, other possible reasons may explain these findings. In fact, at least two analogue miRNAs clusters at 3q25-26 and 17q21 have been described [33].

Of note, all the examined miRNAs, especially those belonging to the same stem loop, showed a quite consistent degree of expression indicating a possible parallel regulation. In this regard, however, it should be noted that when we compared the expression of the single miRNA in CLL samples and reactive lymph nodes, we found hsa-miR-16-1-3p to be more commonly downregulated in cases with 13q deletion. It is unknown, certainly warranting further investigation, whether hsa-miR-16-1-3p downregulation represented a stochastic finding or rather a particular pathobiological relevance. Of note, in this case, it would eventually imply a pathogenic role for an antisense miRNA [41], an occurrence not well characterized yet.

In general, in our series, the percentage of cases presenting with miRNAs downregulation was relatively low. In fact, it was originally reported that hsa-miR-15a/miR-16-1 cluster is downregulated in 75% of CLL, while in our series it was the case in only 40%. Nevertheless, though such similarity might affect the quantification of mature miRNAs, the combined evaluation of stem loops (that conversely are slightly different) allows for avoiding this phenomenon.

It should be noted that it is quite difficult to study deletions by FISH on FFPE cases [42, 43]. Accordingly, the cutoff value defining the presence of 13q del was quite high in our study. However, it is unlikely that this might have affected the interpretation of our results. In fact, when we extended the evaluation of possible correlations between miRNAs level and del13q to all samples, by considering the percentage of cells carrying the 13q deletion, we again failed to detect any significant association.

Finally, as a further analysis, we found that the presence of 13q deletion and lower hsa-miR-16-1-3p expression tended to correlate with the clinical outcome in terms of OS, although the difference was not statistically significant, possibly due to the limited number of cases. Indeed, though this study was not designed (and therefore not powerful) to identify clinical correlates, this is the first confirmation of a possible prognostic impact of hsa-miR-16-1-3p levels after the original studies and the first evidence when tissue samples are considered.

On the other hand, we did not find significant correlation between miRNA expression and any considered pathobiological parameter. This might be again due, on the one hand, to the relatively small sample size. On the other hand, it might be due to the peculiar features of neoplastic cells resident within lymph node that can be somehow different from the circulating ones.

In conclusion, our study explored for the first time the expression profile of the entire miR-15a and miR-16-1 clusters evaluating possible clinicopathological correlation. Of note, as we confirmed the relative independence of miRNA downregulation from the presence of 13qDel, it is reasonable that at least in clinical trials aiming to identify prognostic factors in CLL, miRNAs dosage should accompain conventional genetic studies.

## Supplementary Material

In Supplementary Table 1 you can find a crosstabulation between miRNA regulation and presence of 13q deletion.

## Figures and Tables

**Table 1:** CLL patients.

Samples	Sex	Age	Proliferation center	13q deletion	CD38	ZAP-70	%Ki-67
1	M	60	0	Yes	1	0	15
2	M	81	0	Yes	5	4	2.5
3	M	81	0	Yes	0	3	15
4	M	59	1	Yes	0	5	90
5	M	76	0	Yes	5	n.e	10
6	F	75	1	Yes	0	0	20
7	M	70	1	Yes	0	n.e	17.5
8	M	66	0	Yes	3	5	5
9	F	73	1	Yes	0	n.e	27.5
10	M	63	0	Yes	0	n.e	17.5
11	F	68	1	Yes	0	5	25
12	M	56	0	Yes	0	5	5
13	M	75	1	Yes	4	5	25
14	M	62	1	Yes	0	0	20
15	M	72	1	Yes	5	5	20
16	M	47	1	Yes	2	3	27.5
17	M	62	0	Yes	0	5	55
18	M	58	0	Yes	0	4	15
19	F	56	0	Yes	0	5	5
20	F	73	0	Yes	0	5	5
21	M	70	1	No	0	2	35
22	M	53	0	No	0	4	15
23	F	78	1	No	2	4	17.5
24	M	66	0	No	0	0	2.5
25	M	71	0	No	1	0	50
26	F	75	1	No	0	0	70
27	F	76	0	No	0	4	n.e
28	M	55	0	No	0	5	15
29	M	45	0	No	n.e	4	15
30	F	80	1	No	0	0	35
31	M	74	0	No	0	0	25
32	M	70	0	No	0	1	5
33	M	64	0	No	5	5	15
34	M	58	1	No	0	5	70
35	M	74	0	No	0	1	12.5
36	M	71	1	No	0	4	20
37	F	57	0	No	2	0	60
38	M	55	0	No	0	0	12.5
39	M	79	0	No	4	n.e	n.e
40	F	73	1	No	0	n.e	15

Proliferation center: (1) rich, (0) typical.

CD38 and ZAP70 categories: (1) <10%, (2) 10–15%, (3) 25–50%, (4) 50–75%, (5) >75%.

**Table 2:** Mean features in CLL patients.

	Patients
Sex (male/female)	29/11
Mean age years (range)	67 (45–81)
Higher CD38 expression (score 3–5/tested cases)	7/39
Higher ZAP70 expression (score 3–5/tested cases)	21/34
Mean Ki-67 percentage (range)	24% (2.5%–90%)
Overall survival months median (range)	42 (5–120)

**Table:** (a) Correlations on samples with 13q deletion

	2^−DDCt^_mir16-5p_del	2^−DDCt^_mir15a-5p_del	2^−DDCt^_mir16-1-3p_del	2^−DDCt^_mir15a-3p_del
2^−DDCt^_mir16-5p_del				
Pearson correlation	1	,763**	,843**	,824**
Sig. (2 tailed)		,000	,000	,000
2^−DDCt^_mir15a-5p_del				
Pearson correlation	,763**	1	,969**	,875**
Sig. (2 tailed)	,000		,000	,000
2^−DDCt^_mir16-1-3p_del				
Pearson correlation	,843**	,969**	1	,875**
Sig. (2 tailed)	,000	,000		,000
2^−DDCt^_mir15a-3p_del				
Pearson correlation	,824**	,875**	,875**	1
Sig. (2 tailed)	,000	,000	,000	

**Correlation is significant at the 0.01 level (2 tailed).

**Table:** (b) Correlations on samples without 13q deletion

	2^−DDCt^_mir16-5p_not_del	2^−DDCt^_mir15a-5p_not_del	2^−DDCt^_mir16-1-3p_not_del	2^−DDCt^_mir15a-3p_not_del
2^−DDCt^_mir16-5p_not_del				
Pearson correlation	1	,924**	,746**	,891**
Sig. (2 tailed)		,000	,000	,000
2^−DDCt^_mir15a-5p_not_del				
Pearson correlation	,924**	1	,748**	,936**
Sig. (2 tailed)	,000		,000	,000
2^−DDCt^_mir16-1-3p_not_del				
Pearson correlation	,746**	,748**	1	,864**
Sig. (2 tailed)	,000	,000		,000
2^−DDCt^_mir15a-3p_not_del				
Pearson correlation	,891**	,936**	,864**	1
Sig. (2 tailed)	,000	,000	,000	

**Correlation is significant at the 0.01 level (2 tailed).
