# Supplementary material for: Hsa-miR-15a and Hsa-miR-16-1 Expression Is Not Related to Proliferation Centers Abundance and Other Prognostic Factors in Chronic Lymphocytic Leukemia
Source: Biomed Res Int. 2013 Dec 12;2013:715391. doi: 10.1155/2013/715391 (PMC3874364; doi:10.1155/2013/715391)
Supplement: Supplementary file 1 — In Supplementary Table 1 you can find a crosstabulation between miRNA regulation and presence of 13q deletion. [file 715391.f1.pdf]

**Supplementary Table 1. Crosstabulation between miRNA regulation and presence of 13q deletion**

hsa-miR-16-5p Crosstabulation

| | | Groups | | Total |
|---|---|---|---|---|
| | | Deleted | Not Deleted | |
| miR16-5p | DOWN | 11 | 8 | 19 |
| | UP | 7 | 8 | 15 |
| Total | | 18 | 16 | 34 |

Fisher Exact test Deleted VS Not Deleted p-value= 0,730

hsa-miR-15a-5p Crosstabulation

| | | Groups | | Total |
|---|---|---|---|---|
| | | Deleted | Not Deleted | |
| miR15a-5p | DOWN | 5 | 5 | 10 |
| | UP | 9 | 9 | 18 |
| Total | | 14 | 14 | 28 |

Fisher Exact test Deleted VS Not Deleted p-value=1

hsa-miR-15a-3p Crosstabulation

| | | Groups | | Total |
|---|---|---|---|---|
| | | Deleted | Not Deleted | |
| miR15a-3p | DOWN | 9 | 6 | 15 |
| | UP | 4 | 9 | 13 |
| Total | | 13 | 15 | 28 |

Fisher Exact test Deleted VS Not Deleted p-value=0,151

## hsa-miR-16-1-3p Crosstabulation

| | | Groups | | Total |
|---|---|---|---|---|
| | | Deleted | Not Deleted | |
| miR15a-3p | DOWN | 13 | 2 | 15 |
| | UP | 5 | 15 | 20 |
| Total | | 18 | 17 | 35 |

Fisher Exact test Deleted VS Not Deleted p-value=0
